# Effect of germinated brown rice extracts on pancreatic lipase, adipogenesis and lipolysis in 3T3-L1 adipocytes

**DOI:** 10.1186/1476-511X-13-169

**Published:** 2014-11-03

**Authors:** See Meng Lim, Yong Meng Goh, Wen Bin Kuan, Su Peng Loh

**Affiliations:** Department of Nutrition and Dietetics, Faculty of Medicine and Health Sciences, Universiti Putra Malaysia, 43400 Serdang, Selangor Malaysia; Department of Veterinary Pre-Clinical Science, Faculty of Veterinary Medicine, Universiti Putra Malaysia, 43400 Serdang, Selangor Malaysia; Laboratory of Molecular Biomedicine, Institute of Bioscience, Universiti Putra Malaysia, 43400 Serdang, Selangor Malaysia

**Keywords:** Obesity, Germinated brown rice, Pancreatic lipase, 3T3-L1 adipocytes

## Abstract

**Background:**

This study investigated anti-obesity effects of seven different solvent (n-hexane, toluene, dicholoromethane, ethyl acetate, absolute methanol, 80% methanol and deionized water) extracts of germinated brown rice (GBR) on pancreatic lipase activity, adipogenesis and lipolysis in 3T3-L1 adipocytes.

**Methods:**

GBR were extracted separately by employing different solvents with ultrasound-assisted. Pancreatic lipase activity was determined spectrophotometrically by measuring the hydrolysis of *p*-nitrophenyl butyrate (*p*-NPB) to *p*-nitrophenol at 405 nm. Adipogenesis and lipolysis were assayed in fully differentiated 3T3-L1 adipocytes by using Oil Red O staining and glycerol release measurement.

**Results:**

GBR extract using hexane showed the highest inhibitory effect (13.58 ± 0.860%) at concentration of 200 μg/ml followed by hexane extract at 100 μg/ml (9.98 ± 1.048%) while ethyl acetate extract showed the lowest (2.62 ± 0.677%) at concentration of 200 μg/ml on pancreatic lipase activity. Water extract at 300 μg/ml showed 61.55 ± 3.824% of Oil Red O staining material (OROSM), a marker of adipogenesis. It significantly decrease (p < 0.05) lipid accumulation than control (OROSM = 100%), follow by ethyl acetate extract at 300 μg/ml (OROSM = 65.17 ± 3.131%). All the GBR extracts induced lipolysis with 1.22-1.83 fold of greater glycerol release than control.

**Conclusions:**

GBR extracts especially the least polar and intermediate polar solvent extracts exhibited inhibitory effect on pancreatic lipase, decrease fat accumulation by adipocyte differentiation inhibition, and stimulate lipolysis on adipocytes. Therefore, GBR could be furthered study and developed as a functional food in helping the treatment and/or prevention of obesity.

## Background

Overweight and obesity are chronic metabolic disorder caused by an imbalance between food intake and energy expenditure [1]. Pancreatic lipase, the key lipid digesting enzyme secreted by the pancreatic acinar cells [2], is responsible for the hydrolysis of about 50-70% of total dietary fats [3]. The digested fat is either use as energy source or absorbed and accumulated into adipocytes. Excessive differentiation in the number (hyperplasia) and/or size (hypertrophy) of adipocytes are the characteristics in obesity [4]. Adipocytes are not only fat storage cells but are active endocrine organs secreting adipokines that regulate multiple metabolic homeostasis [5]. Therapeutics for obesity that targeted on these two approaches are lipase inhibition, adipocytes differentiation inhibition and promote lipid metabolism [6]. Although few anti-obesity drugs that are available in the market are meant for therapeutic approach, most of them have adverse side effects [7]. Orlistat (Xenical™), a specific pancreatic lipase inhibitor clinically approved by Food and Drug Administration could block approximately 30% absorption of dietary fat [8]. However, the uses of orlistat have been reported with some adverse effects which include oily stools, diarrhea, flatulence, bloating, abdominal pain, dyspepsia, and fecal spotting [9, 10]. The existing side effects and failure of drugs to continue to be used in long term management, elucidated the need for efficacious yet safe treatments to help prevent disease progression and protect patients from weight regain [11, 12]. Many novel research works have recently come to the fore in discovering potential anti-obesity effect especially from natural sources with the inhibition of fat absorption and/or fat accumulation in the body by interrupting the lipase and adipocyte activity [13–16]. According to Birari *et al*., [3], natural products might be an excellent alternative strategy for the development of safe and effective anti-obesity drugs yet much of them are still unexplored.

Rice (*Oryza sativa* L.) is a major staple food for more than half of the world’s population especially people in Asia and as the second-most consumed cereal grain [17, 18]. It is a main source of energy and accounts for nearly quarter of global energy intake [19]. Germinated brown rice (GBR) is produced by soaking the whole kernel of brown rice in water until it produced a germ of approximately 1mm long [20]. During the process of germination, the chemical compositions of the rice change drastically. All the dormant enzymes are activated to break down the large molecular substances, generating essential compounds and energy [21, 22]. The amount of nutrients such as γ-aminobutyric acid (GABA) [23–25], ferulic acid [19], phytic acid [26], dietary fiber [26], tocotrienols [25], some minerals (magnesium, potassium, zinc), γ-oryzanol [27], prolylendopeptidase inhibitor and antioxidants compounds [28] showed significant increased after germination. The palatability and texture of GBR were also noted to have improved after germination [29]. Research that focus on diminishing fat hydrolysis and their absorption in the gastrointestinal tract through the use of GBR could be an alternative prescription in reducing fat digestion and fat deposition.

In recent times, ultrasound-assisted extraction (UAE) has been extensively applied to extract active compounds from plant materials such as traditional Chinese medicine [30, 31], fruits [32], soy products [33], seed [34], and wheat germ [35]. UAE decrease extraction time and increase extraction yields to minimize problems such as long extraction time and require relatively large quantities of solvent that faced by conventional extraction methods like maceration extraction and soxhlet extraction [30, 36]. In this study, crude extracts of GBR from different polarity solvents with ultrasound-assisted were screened for potential anti-obesity effects by examining their lipase inhibition activity supplemented with their effects on adipogenesis and lipolysis in cultured 3T3-L1 adipocytes.

## Results and discussion

### Ultrasound-assisted extraction yields

Extraction is the initial step in isolating phytochemicals from plant materials [37]. Polarity of solvent and plant matrix will influence the efficiency of extracting the bioactive compounds from the sample [31, 37]. Thus, selection of the most appropriate solvent for extracting bioactive compounds from sample is a crucial process for investigates any experiments. Usually the least polar solvents are considered to be suitable for the extraction of non-polar fraction [38]. A similar principal is applied to semi-polar and polar fraction. Seven different solvents with a wide range of polarities (n-hexane, toluene, dicholoromethane, ethyl acetate, absolute methanol, 80% methanol and deionized water) were used to extract the bioactive compounds of GBR under the same conditions. UAE had been applied in this present study due to the fact that UAE can reduce the extraction time, reduce solvent consumption and give higher yield of bioactive compounds [30, 36].

The results of extraction yields are summarized in Table 1. Overall, water extract (5.91 ± 0.853%) gave the highest extraction yields, followed by toluene (2.68 ± 0.393%) and ethyl acetate (2.61 ± 0.136%). The findings of this study were similar to the oil extraction yield of rice bran by hot air, microwave, roasting, and steaming which were 5.53%, 4.81%, 4.77% and 3.41%, respectively [39]. The reason for higher yield for water extraction might be longer heating time caused excessive swelling of the material such as starch when only water is used as the solvent [40]. The starch in water forms more floccule that could adsorb the effective compounds extracted [40]. Besides, different polarity and viscosity of the solvent used might influence the extraction efficiency [31]. A few studies reported that the additional of small percentages of water to the extraction solvent can sometime help to increase extraction yield [41, 42]. The extract yield of 80% methanol (1.79 ± 0.292%) showed higher yield than absolute methanol (1.20 ± 0.206%) although they were not significantly different (p > 0.05).

**Table 1:** **Extraction yields of different solvent of GBR extracts with ultrasound-assisted**

Type of extracts	Yields (%)
Hexane	2.19 ± 0.657^a,b^
Toluene	2.68 ± 0.393^b^
Dichloromethane	1.71 ± 0.066^a,b^
Ethyl acetate	2.61 ± 0.136^b^
Absolute methanol	1.20 ± 0.206^a^
80% Methanol	1.79 ± 0.292^a,b^
Water	5.91 ± 0.853^c^

^a-b^Values with different lower case letters are significantly different at p < 0.05.

### Pancreatic lipase inhibition

**Figure 1:**
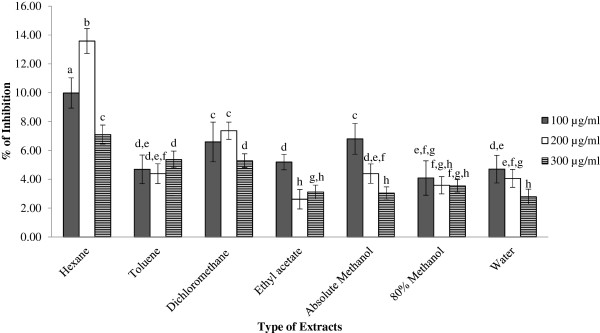
**Effect of GBR extracts on pancreatic lipase activity.** Results are given as a mean value ± S.D. of six replicate measurements. Bar graphs represent the % of inhibition of various GBR extracts in different concentrations on lipase activity. ^a-h^Values with different lower case letters are significantly different at p < 0.05.

Pancreatic lipase inhibition is one of the most widely used models to investigate the potential efficacy of natural products as anti-obesity drugs [3]. Therefore, pancreatic lipase *in vitro* model was used to evaluate the inhibitory effect on pancreatic lipase activity of all the GBR extracts at concentration of 100, 200 and 300 μg/ml by measuring the hydrolysis of *p*-NPB to *p*-nitrophenol. The anti-lipase activity was expressed as percentage of inhibition of control at 0% and is presented in Figure 1. Overall, the inhibitions of pancreatic lipase of various solvent extracts of GBR were ranged from 2.62% to 13.58%. All the extracts did not suppress pancreatic lipase activity in a dose-dependent manner in the concentration of 100, 200, and 300 μg/ml. Extracts at concentration of 100 μg/ml exhibited stronger inhibitory effects than extracts at concentration of 200 μg/ml although some of the extracts were not significantly different (p > 0.05). The inhibitory activities of this study were in line to anti-lipase activities of certain natural plant species found in Korea or Asia [15], with inhibition ranged from 2.5% to 38%.

The hexane extract of GBR showed significantly higher (p < 0.05) inhibitory effect on pancreatic lipase (7.11-13.58%) than other extracts. Some non-polar compounds that present in GBR might play an important role in the lipase inhibition. Rice bran oil contains phytosterols that could be present as intermediate products such as cycloartenol and 24-methylene cycloartanol, as well as ferulic acid esters (oryzanol) [43]. According to Zaburuth *et al*., [44], phytosterols found in hexane extract of *Operculina turpethum* leaves, showed 44.26% of inhibition on pancreatic lipase. Orlistat, as a positive control, showed an IC_50_ of 1.56 μg/ml (y = 10.338ln(x) +45.39, r^2^ = 0.979) in this study on pancreatic lipase activity but no IC_50_ was found for all GBR extracts at concentration up to 300 μg/ml. The lower potency than orlistat suggests that GBR extracts per se might not as effective as commercial drug in inhibiting of pancreatic lipase activity. This is probably due to the activity of a crude extract includes both active and non-active constituents which could be lower than that of the active constituent alone [45].

Inhibitors of pancreatic lipase play an important role in the treatment of obesity. Through gastrointestinal mechanisms, interfering of nutrient digestion and absorption in the body may attempt to decrease the digestion of fat to free fatty acid and monoglycerides that will be further synthesize and accumulate in the adipocytes [3]. Previous studies done on rice bran have also been reported to reduce the absorption of indigested fat by inhibiting the pancreatic lipase activity [46, 47]. GBR is rich in bioactive compounds and has inhibitory effect on pancreatic lipase. This is suggesting that it may be a useful alternative to treat obesity by limiting the dietary fat absorption and accumulation of fat in adipocytes since rice is a daily staple food in some of the countries.

### Adipocyte cell viability

MTT assay was done to assess the cell viability of 3T3-L1 preadipocytes treated with various concentrations of GBR extracts. The cell viabilities were ranged from 73.58% to 108.31% after 72 hr incubation with the GBR extracts (Table 2). The cell viabilities of extracts at the highest concentration (300 μg/ml) were significantly lower (p < 0.05) than the control (without treatment), but the cell viability of 3T3-L1 preadipocytes treated with GBR water extract did not showed significant differences (p > 0.05) than control. It is because the GBR itself might not toxic to the 3T3-L1 preadipocytes, but the solvents that used during extraction might toxic to the cells. In addition, non-cytotoxicity is defined as viability ≥70% compared to untreated cells [48]. All the extracts in different concentrations included the highest concentration (300 μg/ml) were significant higher (p < 0.001) than 70%. Thus, all the extracts at concentration of 0-300 μg/ml were considered as non-cytotoxic to 3T3-L1 preadipocytes.

**Table 2:** **Effects of GBR extracts on preadipocyte viabilities after 72 hr incubation**

Type of extracts	Concentration (μg/ml)						
	0	5	25	50	100	200	300
Hexane	96.30 ± 3.249^a^	92.47 ± 4.040^b^	90.22 ± 1.259^b^	89.76 ± 0.826^b^	86.12 ± 1.663^c^	82.48 ± 3.154^d^	80.60 ± 3.120^d^
Toluene	91.31 ± 2.122^a^	88.02 ± 1.777^b^	81.49 ± 1.547^c^	80.45 ± 1.189^c^	77.54 ± 3.144^d^	76.05 ± 0.585^d^	75.31 ± 1.231^d^
Dichloromethane	90.25 ± 2.571^a^	89.24 ± 1.266^a,b^	88.00 ± 1.977^b^	82.69 ± 0.813^c^	85.76 ± 0.823^d^	87.33 ± 0.886^b,d^	78.87 ± 1.883^e^
Ethyl acetate	90.78 ± 3.005^a^	89.93 ± 1.068^a^	88.89 ± 3.095^a^	88.64 ± 2.193^a^	88.25 ± 1.471^a,b^	85.74 ± 1.726^b^	73.58 ± 1.963^c^
Absolute methanol	98.88 ± 0.565^a^	95.69 ± 2.206^b^	102.83 ± 2.619^c^	107.94 ± 2.020^d^	108.31 ± 1.559^d^	106.22 ± 2.792^d^	105.30 ± 4.343^c,d^
80% Methanol	93.96 ± 1.051^a,b^	91.95 ± 2.077^a,c^	89.37 ± 2.503^c,d^	88.94 ± 0.722^d^	88.96 ± 2.916^d^	94.15 ± 0.747^a,b^	95.84 ± 3.754^b^
Water	91.41 ± 3.032^a,b^	89.14 ± 1.118^b^	89.18 ± 3.000^b^	89.02 ± 0.464^b^	87.97 ± 2.850^b^	90.32 ± 2.763^a,b^	92.88 ± 3.923^a^

^a-e^Values within a row followed by different lower case letters are significantly different at p < 0.05.

### Oil red O staining for intracellular triglycerides

**Figure 2:**
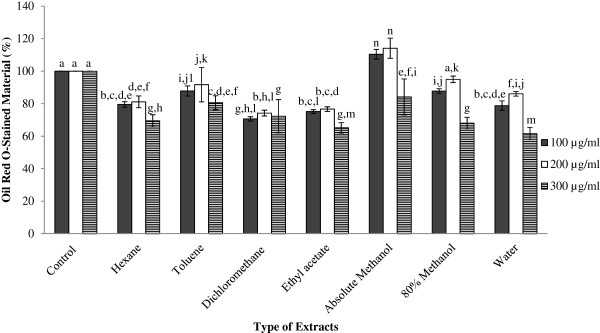
**Relative lipid content quantified via Oil Red O staining in 3T3-L1 adipocytes.** Results are given as a mean value ± S.D. of six replicate measurements. Bar graphs represent the relative amounts of accumulated lipid in cultured 3T3-L1 adipocytes after treated with various GBR extracts in different concentrations. ^a-n^Values with different lower case letters are significantly different at p < 0.05.

Adipose tissue plays an important role in maintaining lipid homeostasis and energy balance by storing triglycerides or liberating free fatty acid in response to changes in energy demands [4]. In Figure 2, the inhibition effects of GBR extracts on fat droplet formation in 3T3-L1 cells, through the quantification method of Oil red O staining, are presented. Oil red O staining material (OROSM) was used as a marker of adipogenesis where the higher the OROSM value, the higher the lipid accumulation inside the cells. Basically, most of the GBR extracts significantly reduce (p < 0.05) lipid accumulation in 3T3-L1 adipocytes compared to control (without treatment), except absolute methanol extract at concentration of 100 μg/ml and 200 μg/ml and 80% methanol extract at concentration of 200 μg/ml.

This present study showed that GBR extracts at concentration of 300 μg/ml showed the highest inhibition in lipid accumulation while extracts at concentration of 100 μg/ml and 200 μg/ml were not significant between each other (p > 0.05) in reducing lipid accumulation of adipocytes. This implied that although GBR extracts at 100-200 μg/ml were able to reduce the lipid accumulation in the adipocytes, GBR extracts at 300 μg/ml showed better inhibition. The active compounds that were present in the extract might inactivate one/some of the pathways in adipogenesis that lead to the decrease lipid accumulation inside the adipocytes.

Besides, the water extract of GBR at concentration of 300 μg/ml exhibited the lowest OROSM with only 61.55 ± 3.824% and followed by ethyl acetate extract at concentration of 300 μg/ml (OROSM = 65.17 ± 3.131%). Meantime, the OROSM of least and intermediate polar solvent extracts are in the range of 65.17% to 91.64% compared to OROSM of polar solvent extracts with OROSM of 61.55% to 114.07%. Lipophilic constituents such as unsaponifiable fraction (phytosterols, triterpene alcohols, 4-methyl-sterols), less polar components (squalene or tocotrienols and α-tocopherol), and γ-oryzanol [49–51] that are present in rice bran might be extracted in the least polar fraction according to their partition coefficient. Alternatively, hydrophilic constituents such as polyphenols, GABA, and reducing sugars are possibly extracted by polar extractant [52, 53]. The active compound(s) in the extract might have inhibitory properties on the adipocytes differentiation.

Interestingly, the OROSM of absolute methanol extract at concentration of 100 μg/ml and 200 μg/ml were higher than the control. The results of the MTT assay also showed that the absolute methanol extract was not only non-cytotoxic to the cell but it increased the cell viability of preadipocytes at these concentrations. This indicated that the compounds extracted by absolute methanol might promote the growth of the adipocytes. These results were contradicted with the study done by Ho *et al*., [54] which used methanol extraction on GBR, reported 49% of inhibition in reduces the OROSM. Further studies are needed to investigate and identify compounds that could contribute to this observation.

### Glycerol level

**Figure 3:**
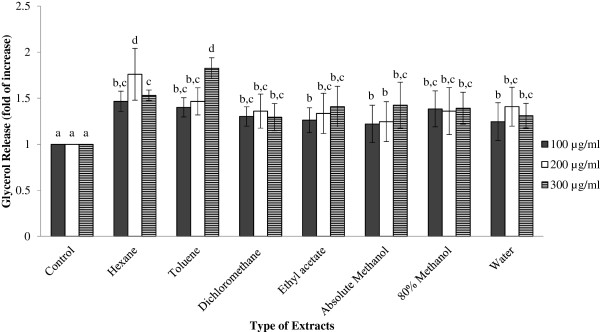
**Extent of lipolysis based on the amount of glycerol released across different GBR extracts.** Results are given as a mean value ± S.D. of six replicate measurements. Bar graphs represent the relative glycerol release content of 3T3-L1 adipocytes after treated with various GBR extracts in different concentrations. ^a-d^Values with different lower case letters are significantly different at p < 0.05.

Lipolysis is a catabolic process that hydrolyzes stored triglycerides in adipose tissue to release free fatty acid and glycerol. Thus, lipolysis of fat cells can regulate the homeostasis of energy by controlling the release of fatty acids and glycerol into plasma [55]. The lipolytic effect of GBR extracts was investigated through the measurement of glycerol released in culture medium after 24 h incubation, as shown in Figure 3. All the GBR extracts stimulated mild lipolysis by induced 1.22-1.83 fold greater release of glycerol into the culture medium than control. Toluene extract at concentration of 300 μg/ml showed the highest glycerol release content of adipocytes with 1.83 fold of increase and followed by hexane extract at concentration of 200 μg/ml (1.76 fold). Absolute methanol extract at concentration of 200 μg/ml showed the lowest glycerol release content of adipocytes with 1.22 fold of increase than control.

Lipolysis in adipocytes is known to be signaled by the activation of intracellular cAMP level, which in turn activates protein kinase A and substrates such as hormone-sensitive lipase and perilipin [56]. Hormone-sensitive lipase is a key enzyme in the mobilization of free fatty acids from adipocytes [56]. According to Ho *et al*., [54], hormone-sensitive lipase was found to be up-regulated in mice group that administrated with GBR and hypothesized that vitamin E, oryzanol and GABA were the active compounds that improve lipid metabolism in obese mice. This hypothesis is line with this present study where the least polar solvents extracts showed slightly better lipolytic effect than polar solvent extracts.

## Conclusions

In conclusion, GBR extracts especially least polar and intermediate polar solvent extracts showed inhibition on pancreatic lipase, suppress adipogenesis, and stimulate lipolysis in 3T3-L1 adipocytes. This suggested that GBR may have nutraceutical potential in the treatment and/or prevention of obesity. Further study is needed to evaluate the anti-obesity effect of GBR in experimental animals and human study.

## Methods

### Chemicals and reagents

Dimethyl sulfoxide (DMSO), lipase (Type II; from Porcine pancreas), *p*-nitrophenyl butyrate (*p*-NPB), potassium phosphate monobasic (KH_2_PO_4_), potassium phosphate dibasic (K_2_HPO_4_), orlistat, 3-(4,-dimethy-lthiazol-2-yl)-2,-diphenyl-tetrazoliumbromide (MTT), insulin, dexamethasone, 3-isobutyl-1-methyl-xanthine (IBMX), Oil Red O, and phosphate-buffered saline (PBS) were purchased from Sigma-Aldrich (St. Louis, MO, USA). n-Hexane, toluene, dicholoromethane, ethyl acetate, methanol, and isopropanol were purchased from Merck (Darmstadt, Germany). Dulbecco’s modified Eagle’s medium (DMEM), bovine calf serum (BCS), foetal bovine serum (FBS), and penicillin/streptomycin were purchased from GIBCO (BRL Life Technologies, Grand Island, NY, USA). The water was obtained from Milli-Q water purification system (Millipore, Bedford, MA, USA). All chemicals and reagents used in the study were of analytical grade.

### Sample preparation

GBR (*Oryza sativa* L.) was provided by Laboratory of Molecular Biomedicine, Institute of Bioscience, Universiti Putra Malaysia (Selangor, Malaysia). The GBR was grounded to fine powder, passed through the 20-mesh sieve, and stored at -20°C in a tightly sealed plastic bag until further analysis.

### Ultrasound-assisted extraction

The GBR was extracted according to method by Boonsiripiphat *et al*., [57] with modification using a sonicator. GBR (30 g) was mixed with 100 ml of hexane in a ratio of about 1:3 (w/v). The mixture was immersed into the ultrasonic bath (Power Sonic 405, Hwashin Technology, Korea) equipped by a generator with an output of 350W and input of AC 230V and 40 kHz, and kept for sonication for 30 min with water bath temperature maintained at 40°C. The extract was then centrifuged (Rotofix 32A, Hettich Zentrifugen, Germany) at 3500 rpm for 10 min to obtain the supernatant and the rest was re-extracted for another 2 times under the same conditions. The combined filtrate was filtered through Whatman No.1 filter paper (Whatman International, England) and evaporated with a rotary evaporator (Büchi Rotavapor R-200, Büchi Labortechnik AG, Switzerland) below 40°C. The procedures of extraction were repeated in the same manner except different extraction solvents (toluene, dicholoromethane, ethyl acetate, absolute methanol, 80% methanol and deionized water) were used. The extracts were stored at -20°C until analysis. The yield of extraction was calculated as follows:


where:

DWe = dry weight of sample extract after evaporation of solvent.

DWs = dry weight of the sample powder.

The GBR extracts were dissolved in DMSO at a final concentration of 0.1% prior use.

### Pancreatic lipase inhibition assay

The activity of porcine pancreatic lipase (type II) was colorimetrically evaluated through the measurement of the release of *p*-nitrophenol. The method used for measuring the pancreatic lipase activity was modified from Slanc *et al*., [58] and Zheng *et al*., [13]. Pancreatic lipase stock solutions (1 mg/ml) were prepared in a 0.1M potassium phosphate buffer (pH 6.8) and the solutions were stored at -40°C. A 10 mM solution of *p*-NPB as a substrate was prepared in acetonitrile. Absolute ethanol was then added to reach a final concentration of 3.33 mM of *p*-NPB. Briefly, 12 μl of enzyme buffer was added to 162 μl of potassium phosphate buffer (0.1M, pH 7.2, 0.1% Tween 80). Either 16 μl of the extracts or orlistat as a positive control was then mixed into the solution and incubated for 30 min at 37°C. Subsequently, 10 μl of substrate solution was added and the enzymatic reactions were allowed to proceed for another 30 min at 37°C. The amount of *p*-nitrophenol release in the reaction was measured at 405 nm using a microplate reader (SIRIO S SEAC, Italy). The absorbance reading was compared to the control, which contained same amount of buffer solution, instead of the extract. The inhibitory activity (%) was calculated according to the following formula:


where:

 = absorbance without extract

 = absorbance with extract.

### Cell culture and differentiation

3T3-L1 preadipocytes were obtained from the American Type Culture Collection (Manassas, VA, USA). The cells were maintained in DMEM high glucose containing 10% (v/v) BCS and penicillin/streptomycin (100 units/ml, 100 μg/ml, respectively) at 37°C in a humidified atmosphere of 95% air and 5% CO_2_. Fresh medium were provided to the cells every 2-3 days till cells were about 80% confluence. As described previously by Ahn *et al*., [59], 3T3-L1 preadipocytes were seeded into 24-well plates at a density of 2×10^4^ cells/well to differentiate preadipocytes into adipocytes and grown to confluent in three days. At this point (referred to as day 0), preadipocytes were stimulated for 3 days with a differentiation medium (10% FBS/DMEM medium supplemented with 0.5 mM IBMX, 1.0 μM dexamethasone and 1.0 μg/ml insulin). On day 3, the medium was replaced with maturation medium (10% FBS/DMEM medium and 1.0 μg/ml insulin) for an additional 2 days. On day 5, the culture medium was replaced again with only 10% FBS/DMEM, which were changed every two days until day 9. At this time, the cells exhibited a lipid-filled phenotype, which is characteristic of mature adipocytes.

### Treatment of GBR extracts

To investigate the effects of GBR extracts on adipogenic differentiation, differentiating 3T3-L1 cells were treated with GBR extracts at various concentrations with medium changed every times from day 0 to day 7. The control was treated with same medium without GBR extracts.

### Adipocyte cell viability assay

MTT assay was performed to investigate the cell viability of 3T3-L1 preadipocytes according to method developed by Mosmann [60]. The cell were seeded into 96-well plate at a density of 1×10^4^ cells/well in DMEM medium and allowed to attach for 24 hr. After that, the culture medium was replaced with 200 μl (0, 5, 25, 50, 100, 200, 300 μg/ml) of GBR extracts in DMEM medium. The cells were incubated for 72 hr and untreated cells served as control. Ten microliters of MTT solution (5 mg/ml) in PBS (pH 7.4) was added to each well. After 3 hr, the unreacted dye was removed and the formed formazan crystals were dissolved in 100 μl/well DMSO. After a few minutes at room temperature to ensure that all crystals were dissolved, the plates were read on FLUOstar Omega plate reader (BMG Labtech, Offenburg, Germany), using a test wavelength of 570 nm, a reference wavelength of 630 nm. The cell viability (%) was expressed as the percentage of cell viability compared to the control and calculated according the following formula:


where:

 = absorbance of tested sample extract.

 = absorbance without tested sample extract.

### Oil Red O staining for intracellular triglycerides

Intracellular lipid accumulation in 3T3-L1 adipocytes was measured using Oil-red O that previously described by Ramírez-Zacarías *et al*., [61]. Briefly, the cells were washed gently twice with ice-cold PBS (pH 7.4) and fixed with 10% formalin at room temperature for at least 30 min to 1 hr in room temperature. After removal of the 10% formalin, wells were washed with 60% isopropyl alcohol for 5 min and then washed exhaustively with PBS. Wells were allowed to dry completely before the addition of filtered Oil Red O solution for 30 min at room temperature. The staining of lipid droplets in 3T3-L1 adipocytes were exhaustively rinsed three times with PBS. Stained oil droplets were extracted with 100% isopropanol for 10 min to quantify intracellular lipids. The extracted dye was then immediately removed by gentle pipetting and its absorbance was measured spectrophotometrically at 520 nm using FLUOstar Omega plate reader (BMG Labtech, Offenburg, Germany). The Oil Red O-stained material (OROSM, %) was compared to control wells containing cell culture medium without the GBR extract and calculated according the following formula:


where:

 = absorbance of tested sample extract.

 = absorbance without tested sample extract.

### Measurement of glycerol

Lipolysis assay was performed to evaluate the possible lipolytic activity of GBR extracts by measuring their ability to release glycerol from differentiated 3T3-L1 cells. 3T3-L1 preadipocytes were induced to adipocytes as described previously. On day 10, cells were washed with filtered Hank’s Balanced Salt solution and then treated with GBR extracts for 24 hr in filtered Hank’s Balanced Salt solution with 2% bovine serum albumin. The glycerol level secreted by adipocytes was determined using enzymatic assay kit from Millipore Cat. No. OB 100 (Bedford, MA, USA). Absorbance was determined at 540 nm in a FLUOstar Omega plate reader (BMG Labtech, Offenburg, Germany). A standard curve was prepared, using a standard solution of glycerol at concentration of 0-104 μg/ml (y = 0.0076x +0.0156, r^2^ = 0.998). Glycerol release was then expressed as fold increase, as compared to control for comparison purpose.

### Statistical analysis

Data analyzed by using software IBM SPSS version 21.0. One-way analysis of variance (ANOVA), followed by Duncan was applied to test for differences between groups. One-Sample *T*-Test (two tails) was performed to test for cell viability against 70%. Significant differences were taken at p < 0.05.

## Abbreviations

BCS: Bovine calf serum
DMEM: Dulbecco’s modified Eagle’s medium
DMSO: Dimethyl sulfoxide
FBS: Foetal bovine serum
GABA: γ-aminobutyric acid
GBR: Germinated brown rice
IBMX: 3-isobutyl-1-methyl-xanthine
K_2_HPO_4_: Potassium phosphate dibasic
KH_2_PO_4_: Potassium phosphate monobasic
MTT: 3-(4,-dimethy-lthiazol-2-yl)-2,-diphenyl-tetrazoliumbromide
PBS: Phosphate-buffered saline
*p*-NPB: *p*-nitrophenyl butyrate
S.D.: Standard deviation
UAE: Ultrasound-assisted extraction.
